# Exploring the thalamus L-sign: initial findings and associations with white matter injury in premature infants

**DOI:** 10.1007/s00247-024-05976-8

**Published:** 2024-07-06

**Authors:** Goni Merhav, Sarit Ravid, Mika Shapira Rootman

**Affiliations:** 1 Medical Imaging Division, Rambam Health Care Campus, HaAliya HaShniya 8, PO Box 9602, 3109601 Haifa, Israel; 2 Pediatric Neurology Unit, Rambam Health Care Campus, Haifa, Israel; 3 Ruth and Bruce Rappaport Faculty of Medicine, Technion, Haifa, Israel

**Keywords:** Magnetic resonance imaging, Periventricular leukomalacia, Premature infant, Thalamus, White matter

## Abstract

**Background:**

The thalamus L-sign, characterized by damage to the lateral and posterior parts of the thalamus, has recently been identified as a potential marker of partial prolonged hypoxic-ischemic injury (HII). Although prematurity-related thalamic injury is well documented, its association with the thalamus L-sign is infrequently described.

**Objective:**

The primary objective of this study was to further investigate the thalamus L-sign in premature birth and white matter injury.

**Materials and methods:**

A retrospective analysis of 246 brain magnetic resonance imaging (MRI) scans from preterm infants born before 37 weeks of gestation was conducted to explore the occurrence, characteristics, and associations of the thalamus L-sign with white matter injury.

**Results:**

The L-sign was detected in 12.6% of patients with periventricular leukomalacia (PVL), primarily in severe cases (57.9% of severe PVL). All cases were associated with posterior parieto-occipital PVL. Four patients exhibited unilateral or asymmetric L-signs, which were linked to high-grade intraventricular hemorrhage (IVH) or periventricular hemorrhagic infarction on the ipsilateral side, with the most severe white matter injury occurring on that side. No significant differences were observed regarding gestational age at birth, duration of neonatal intensive care unit hospitalization, percentage of IVH, hypoglycemia, or jaundice between patients with moderate-to-severe PVL with and without the thalamus L-sign.

**Conclusion:**

The thalamus L-sign may serve as a marker for severe parieto-occipital PVL and may be exacerbated and appear asymmetric in cases of ipsilateral IVH or periventricular hemorrhagic infarction.

**Graphical Abstract:**

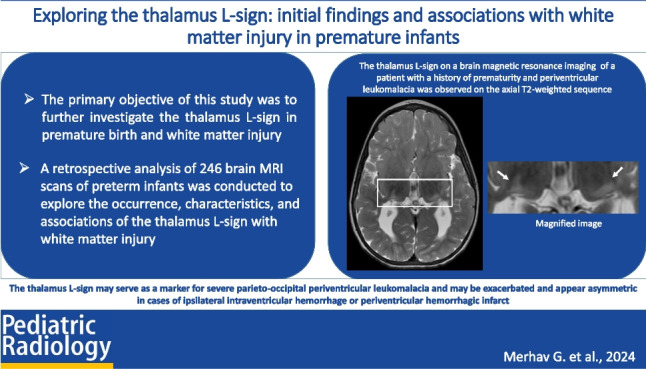

## Introduction

The thalamus L-sign, characterized as an injury limited to the posterior and lateral margins of the thalamus, has recently emerged as a potential marker of partial, prolonged hypoxic-ischemic injury (HII) in term neonates, primarily when involving the posterior watershed region and potentially exacerbated by hypoglycemia [1]. The L-shaped characteristic is likely linked to an irreversible injury of the pulvinar nucleus, lateral geniculate body, and thalamic reticular nucleus, which abuts the posterior limb of the internal capsule [1].

Periventricular leukomalacia (PVL) is the predominant parenchymal injury among survivors of prematurity, leading to significant neurodevelopmental challenges [2–5]. Thalamic injury, primarily exhibiting volume loss, has also been documented in the literature and is frequently detected in brain imaging studies [6–10]. A more selective vulnerability of the pulvinar has also been reported and is associated with posterior parieto-occipital white matter damage, which may lead to cognitive and visual-spatial impairments in preterm infants due to damaged thalamocortical connections between the pulvinar and parieto-occipital cortices [10, 11].

Although recently identified in the literature as a potential marker for differentiating PVL and neurometabolic diseases [12], the thalamus L-sign has rarely been discussed regarding its occurrence in premature infants with PVL, especially concerning the involvement of both the posterior and lateral margins of the thalamus on brain imaging. Additionally, white matter damage in premature infants may also be associated with intraventricular hemorrhage (IVH) and periventricular hemorrhagic infarction. However, the relationship between these conditions and the thalamic L-sign remains unclear.

This study aimed to further explore the characteristics of the thalamus L-sign in relation to prematurity including the severity of white matter injury, patterns of asymmetric L-sign, its association with periventricular hemorrhagic infarction, and additional demographic characteristics.

## Materials and methods

### Ethics approval and study design

This study received approval from the local ethics committee, which granted a waiver for informed patient consent. We retrospectively analyzed brain MR imaging studies from patients born prematurely (before 37 weeks of gestation), focusing on identifying signs of white matter and thalamic injuries. To maintain data integrity, patients with incomplete medical records or unsatisfactory imaging quality were excluded.

### Imaging protocol and equipment

All MR imaging was performed beyond the acute phase of injury, utilizing 1.5-tesla (T) (Siemens, Erlangen, Germany) or 3-T MRI (GE Healthcare, [Waukesha, WI] and Siemens [Erlangen, Germany]) scanners.

The standardized imaging protocol included the following parameters: sagittal T2-weighted fast spin-echo (FSE) images with a slice thickness of 3 mm, repetition time (TR) of 6350 ms, and echo time (TE) of 100 ms. Axial diffusion-weighted imaging (DWI) was performed with a slice thickness of 3 mm, TR of 8000 ms, TE of 65 ms, and a *b*-value of 1000 s/mm^2^, accompanied by apparent diffusion coefficient (ADC) maps. Axial T1 fluid-attenuated inversion recovery (FLAIR) images were acquired with a slice thickness of 3 mm, TR of 2500 ms, and TE of 25 ms. For axial T2-weighted FSE imaging, the slice thickness was 3 mm, with a TR of 5700 ms and TE of 105 ms. Axial susceptibility-weighted angiography (SWAN) images were captured with a slice thickness of 2 mm, TR of 38 ms, and TE of 23 ms. Finally, 3-dimensional (D) sagittal T2 FLAIR-Cube images, including reformats, were obtained with a slice thickness of 1.5 mm, TR of 7000 ms, TE of 100 ms, and an inversion time (TI) of 1950 ms. These parameters were optimized to enhance image quality and diagnostic accuracy across the different imaging sequences.

### Data collection and image analysis

**Fig. 1:**
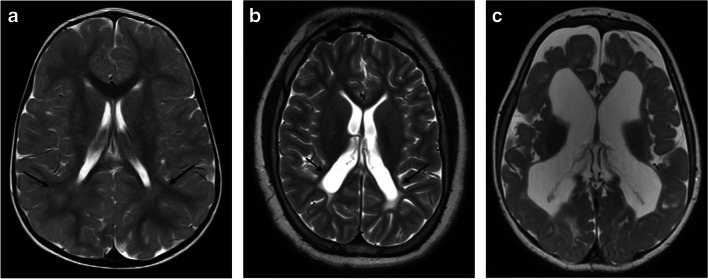
Grading of periventricular leukomalacia (PVL). Axial T2-weighted images of mild (**a**), moderate (**b**), and severe (**c**) PVL. **a** Mild PVL with subtle hyperintensity in the bilateral periventricular white matter adjacent to the atria (*arrows*) in a 19-month-old girl born at 31 weeks gestational age (GA). **b** Moderate PVL in a 14-year-old boy born at 27 weeks GA, characterized by abnormally high signal intensity in the periventricular white matter, as well as ventricular irregularity and mild dilatation (*arrows*). **c** Severe PVL associated with ventriculomegaly in an 11-month-old girl born at 27 weeks GA

**Fig. 2:**
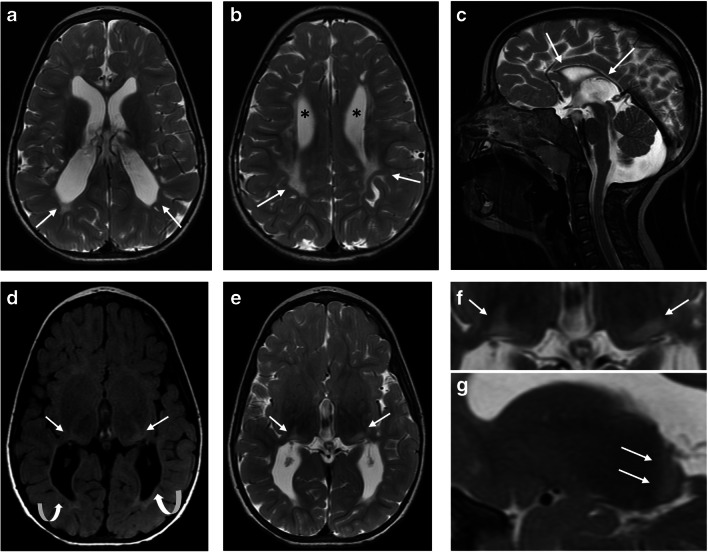
Magnetic resonance imaging findings consistent with periventricular leukomalacia (PVL) and the thalamus L-sign in a 2-year-old boy born at 24 + 1 gestational age (GA). **a** Axial T2-weighted image at the level of the lateral ventricles reveals white matter injury with a predominant posterior distribution, featuring volume loss and gliosis. The lateral ventricles are dilated, displaying the characteristic “wavy” configuration (*arrows*). **b** Axial T2-weighted image captured at a slightly higher level also reveals white matter injury, with volume loss and hyperintensity consistent with gliosis (*arrow*). The lateral ventricles are dilated (*asterisks*), and there are signs of mild delayed myelination. **c** A sagittal midline T2-weighted image shows diffuse thinning of the corpus callosum (*arrows*), resulting from the white matter injury. **d**, **e** Axial fluid-attenuated inversion recovery (**d**) and T2-weighted (**e**) images at the level of the thalami illustrate the “L-sign” with signal changes in the posterior thalami, including the pulvinar and lateral geniculate body, as well as laterally adjacent to the posterior limb of the internal capsule (*arrows*). There is “ex vacuo” dilatation of the posterior lateral ventricles (*curved arrows* in **d**) with mild gliotic changes in the periventricular white matter. Again, note the characteristic wavy configuration of the lateral ventricles. **f** Axial T2-weighted magnified view of the thalami, showing the signal hyperintensity in the posterior and lateral thalami consistent with the “L-sign” (*arrows*). **g** Right parasagittal T2-weighted image showing the T2 hyperintensity of the posterior thalami (*arrows*)

**Fig. 3:**
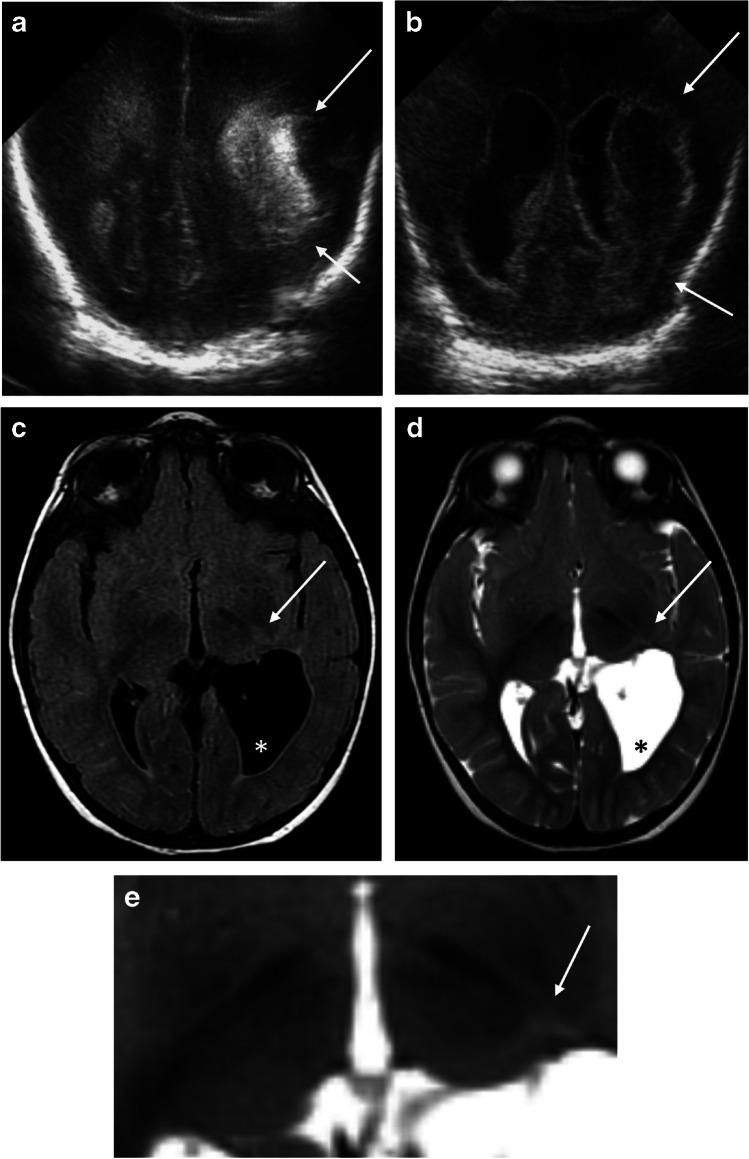
Asymmetric thalamus L-sign in a premature infant girl born at 25 + 6 gestational age (GA). **a** Brain ultrasound in the coronal plane performed at 5 days of life demonstrates increased echogenicity in the left parietal periventricular and deep white matter (*arrows*), consistent with a left periventricular hemorrhagic infarction. Milder echogenicity in the periventricular and deep white matter is also noted on the right side. **b** A follow-up brain ultrasound in the coronal plane at 2 weeks of age reveals the evolution of the hemorrhagic infarct (*arrows*) with reduced echogenicity with wider ventricles. **c**, **d** Axial fluid-attenuated inversion recovery (**c**) and T2-weighted (**d**) brain magnetic resonance images performed at 2 years of age show dilation of the left posterior horn of the lateral ventricle (*asterisks*). This change is secondary to a prior hemorrhage that led to the formation of a porencephalic cyst, which has now become part of the ventricular system. There is more evident white matter volume loss on the left. The thalamus “L-sign” (*arrow*) is only evident on the left. **e** Magnification of (**d**), the axial T2 image, again showcases the thalamus “L-sign” (*arrow*)

Two certified neuroradiologists (M.S.R. and G.M. with 11 and 2 years of experience, respectively) reviewed the imaging studies in consensus, evaluating for signs of PVL and classifying as mild, moderate, or severe according to the criteria described by Choi et al. [2]. Accordingly, grade 1 (mild) PVL is defined as an abnormally high signal intensity in the periventricular white matter on T2-weighted and T2 FLAIR images, most commonly observed bilaterally in the trigone regions of the lateral ventricles. Grade 2 (moderate) PVL refers to periventricular white matter hyperintensity with ventricular wall irregularity, and grade 3 (severe) PVL is identified by diffuse PVL and ventricular dilatation (Fig. 1). PVL was also classified according to the areas involved (anterior, parieto-occipital). Other prematurity-related injuries were also evaluated, including IVH, periventricular hemorrhagic infarction, and post-hemorrhagic hydrocephalus. The thalamus was examined for the L-sign, which indicates injury to the posterior and lateral margins, as shown in Fig. 2. This sign was categorized as either bilateral symmetric or asymmetric, with the latter also indicating which side is more affected (Fig. 3). Retrospective analysis of brain ultrasound images during the acute phase was conducted for cases with an asymmetric thalamus L-sign.

Patient medical records provided additional data, including gestational age at delivery, gender, duration of hospitalization in the neonatal intensive care unit (NICU), presence of hypoglycemia, presence of jaundice, and late complications (cerebral palsy, developmental delay, seizures).

### Statistical analysis

Statistical analysis primarily involved descriptive statistics to summarize the data collected, including calculation of means for continuous variables and percentages for categorical variables. To determine the significance of differences between groups (e.g., between patients with and without the thalamus L-sign, and among different severities of PVL), the chi-square test was used for categorical variables, and the Student’s *t*-test was applied for continuous variables. A *P-*value less than 0.05 was considered indicative of statistical significance. The SPSS statistics software, version 25.0 (IBM Corp., Armonk, NY) was used for subsequent analysis.

## Results

Our review encompassed 246 brain MR studies from patients aged 11 weeks to 15 years, including 103 females. Gestational ages at birth ranged from 24.1 to 36.6 weeks, with a mean of 33.5 weeks. PVL was identified in 103 (41.8%) cases, classified as mild in 45 (43.7%), moderate in 39 (37.9%), and severe in 19 (18.4%).

### The thalamus L-sign

Among the patients with PVL, 13 (12.6%) exhibited the thalamus L-sign: two with moderate PVL and 11 with severe PVL (84.6% of those with the L-sign). Notably, no cases of the thalamus L-sign were detected in patients without parieto-occipital PVL, highlighting its specific association with this condition. A significant difference in the prevalence of the thalamus L-sign was observed, occurring in 5.1% of patients with moderate PVL (two out of 39) and 57.9% of those with severe PVL (11 out of 19), indicating a strong association (*P* < 0.05).

### Asymmetric thalamus L-sign

Four cases of the thalamus L-sign were observed to be either unilateral or asymmetric. A summary of the characteristics of these cases is provided in Table 1. Detailed analysis of early brain ultrasound scans in these cases revealed either IVH grade III or periventricular hemorrhagic infarction ipsilateral to the thalamus changes. Furthermore, periventricular white matter injury was also asymmetric and more pronounced on the same side. The mean gestational age at birth for this group was 27.3 weeks, significantly lower than the 32.5 weeks observed in patients with symmetric thalamus L-sign presentations (*P* < 0.05).

**Table 1:** Characteristics of asymmetric or unilateral thalamus L-sign in premature infants with white matter injury

Patient	Gestational week at birth	PVL	Thalamus L-sign	Intraventricular hemorrhage	White matter injury
1	27	Severe	Asymmetric Left > right	Right grade IV PVHI Left grade III	Left > right
2	25	Moderate	Unilateral left	Left grade IV PVHI	Left > right
3	29	Moderate	Unilateral right	Right grade III	Right > left
4	26	Severe	Unilateral left	Right grade II Left grade IV PVHI	Left > right

*PVHI* periventricular hemorrhagic infarct, *PVL* periventricular leukomalacia

### Clinical outcomes

Comparison between patients with moderate to severe PVL, with and without the thalamus L-sign, showed no significant differences in gestational age at birth, neonatal intensive care unit hospitalization duration, percentage of IVH, hypoglycemia, or jaundice (Table 2). Follow-up in the neurology clinic revealed that nine children with the thalamus L-sign experienced seizures (69.2%), 10 had developmental delays (76.9%), and 11 were diagnosed with cerebral palsy (84.6%).

**Table 2:** Clinical and demographic characteristics of periventricular leukomalacia patients with and without the thalamus L-sign

	Moderate to severe PVL, absent thalamus L-sign (*n*=45)	Moderate to severe PVL with thalamus L-sign (*n*=13)	*P-*value
Gestational age at birth (weeks)	31	30	0.15
Time of hospitalization in the NICU	23	22	0.34
Percentage of IVH	10	4	0.71
Hypoglycemia	10	4	0.71
Jaundice	17	6	0.74

*IVH* intraventricular hemorrhage, *NICU* neonatal intensive care unit, *PVL* periventricular leukomalacia

## Discussion

In this study, we investigated the incidence of the thalamus L-sign on brain MRI scans in a cohort of patients with a history of prematurity. Our analysis revealed that the L-sign, a radiological feature characterized by changes in the posterior and lateral margins of the thalamus, was notably associated with posterior parieto-occipital PVL. This association was particularly strong in cases of severe PVL, wherein the L-sign was detected with the greatest frequency. Notably, in our cohort, an asymmetric presentation of the L-sign was documented in four cases. Each of these cases had experienced a preceding ipsilateral IVH of grade III or a periventricular hemorrhagic infarction, accompanied by more pronounced white matter injury on the affected side.

Prematurity-related thalamic injury is a well-known phenomenon and may manifest through secondary mechanisms, involving anterograde or retrograde (trans-synaptic) damage, or via direct damage to the thalamic structures, as evidenced by neuropathological and radiological investigations revealing volumetric reduction and gliosis [9, 13–15]. The thalamus L-sign, along with its link to posterior parieto-occipital parenchymal damage, suggests that these injuries primarily occur as secondary effects rather than direct insults to the thalami. Advanced neuroimaging techniques, including functional MRI and diffusion tensor imaging, have clarified this relationship by highlighting the specialized connectivity between the posterior-lateral thalamic regions and the parieto-occipital cortex [16]. This connectivity is crucial for visual processing and spatial attention, and disruptions can result in impairments in visual perception, spatial awareness, and attentional control [11].

As previously described by Misser et al. [1], the thalamus L-sign may be indicative of parieto-occipital cortical injury, which is observable in situations of partial prolonged HII and further exacerbated by additional hypoglycemia-induced parenchymal damage. In the context of prematurity, thalamic alterations mirror damage to the white matter tracts connecting the thalamus to the parieto-occipital cortices. Consequently, an expanded interpretation of the thalamus L-sign could indicate damage to the posterior thalamocortical connections, potentially manifesting as injuries within both gray and white matter.

Four cases exhibited an asymmetric thalamic L-sign, which was exacerbated by additional white matter injury related to IVH and periventricular hemorrhagic infarction. IVH typically originates from the germinal matrix, and as the germinal matrix bleed enlarges, the underlying ependyma breaks, filling the ventricle with blood. The severity of IVH is graded according to ultrasound findings, with grades ranging from hemorrhage confined to the germinal matrix (grade I) to IVH with acute ventricular dilatation (grade III). Parenchymal echogenicity is often referred to as grade IV IVH; however, such lesions usually represent periventricular hemorrhagic infarction, which results from compression of the medullary veins draining the white matter rather than an intraventricular hemorrhage that extends to the parenchyma. IVH may cause damage to the adjacent white matter, as revealed by autopsy studies showing evidence of white matter injury in 50–80% of infants with IVH [17–20]. Typical microscopic findings in the periventricular white matter and germinal matrix include scarring and astrogliosis with reactive astrocytes, hemosiderin-laden macrophages, and calcifications [19–21]. Periventricular hemorrhagic infarction on the other hand usually evolves into a single porencephalic cyst that communicates with the lateral ventricle or a cluster of periventricular cysts that remain separate from the ventricle. Hemorrhagic necrosis in periventricular hemorrhagic infarction is typically unilateral and asymmetric, resulting in asymmetric white matter injury [20]. Therefore, the presence of an asymmetric thalamic L-sign signifies an asymmetric white matter injury, probably related to additional ipsilateral high-grade IVH. This contrasts with the exaggerated L-sign described in term infants with partially prolonged HII and hypoglycemia, which typically affects the bilateral posterior parieto-occipital cortex and adjacent white matter in a symmetric manner.

Although further studies of the thalamus L-sign with long-term follow-up are warranted, our findings suggest that it may aid in prognostication and serve as a marker for future disabilities, including the development of cerebral palsy. Early identification of the L-sign may therefore facilitate timely, targeted interventions, aligning with current recommendations to begin treatment at the diagnosis of cerebral palsy or high-risk cerebral palsy [22].

From an imaging perspective, radiologists should be familiar with the thalamus L-sign and its significance. Recognizing the L-sign can also help avoid misdiagnoses and irrelevant considerations, such as neurometabolic diseases, thereby improving diagnostic accuracy and patient management.

This study has several limitations that require consideration. First, the limited number of patients in the cohort restricts our ability to further investigate the thalamus L-sign in prematurity, particularly in cases where it is asymmetric (with only four patients in this study). Another limitation of this study is that the images were interpreted in consensus rather than independently. This approach, while ensuring a thorough and comprehensive evaluation, limits the ability to quantify interobserver variability. An additional limitation of our study was that patients were evaluated retrospectively, with a lack of standardized recording of clinical information and inconsistency in the timing and protocols of MRI studies. Furthermore, the absence of advanced MRI techniques, such as diffusion tensor imaging, restricts a comprehensive evaluation of the white matter tracts, which is crucial for gaining thorough neurodiagnostic insights. Finally, the absence of long-term follow-up data hinders our understanding of the prolonged neurocognitive consequences of the thalamus L-sign. Future studies aimed at further exploring the thalamus L-sign could significantly enhance our understanding of this finding. This includes examining the thalamus L-sign in other populations, such as term neonates with white matter injury and PVL. Additionally, future prospective studies with larger cohorts, standardized protocols utilizing advanced techniques, and long-term clinical follow-up could help us further characterize the thalamus L-sign and its clinical significance.

## Conclusion

The thalamus L-sign, previously identified in cases of partial prolonged HII, can also manifest in late MRI scans of patients with a history of prematurity and posterior parieto-occpital PVL, especially when severe. Additional white matter injury on the ipsilateral side, resulting from IVH or periventricular hemorrhagic infarction, may exacerbate the “L-sign,” leading to asymmetry in these instances. The L-sign likely indicates damage to the thalamo-cortical circuit, encompassing both gray matter injury, as observed in HII, and white matter injury associated with prematurity.

## Data Availability

The data that support the findings of this study are available from the corresponding author upon reasonable request.
